# Platelets in cancer and immunotherapy: functional dynamics and therapeutic opportunities

**DOI:** 10.1186/s40164-025-00676-x

**Published:** 2025-06-13

**Authors:** Changjing Cai, Yiting Liu, Ruohuang Lu, Xudong Fan, Shan Zeng, Pingping Gan

**Affiliations:** 1 Department of Oncology, Xiangya Hospital, Central South University, Changsha, 410008 Hunan China; 2 National Clinical Research Center for Geriatric Disorders, Xiangya Hospital, Central South University, Changsha, 410008 Hunan China; 3 Department of Stomatology, the Third Xiangya Hospital, Central South University, Changsha, 410013 Hunan China

**Keywords:** Cancer, Platelet, Tumor microenvironment, Immunotherapy

## Abstract

Platelets play a critical role in tumor immunity, particularly in promoting cancer progression. Numerous studies suggest that platelets could serve as a novel target for cancer immunotherapy, however, no comprehensive reviews have yet summarized and discussed this potential. Our review provides an in-depth discussion of the roles and mechanisms of platelets within both the immunosuppressive tumor microenvironment and the anti-tumor immune microenvironment. Additionally, we summarize the key therapeutic targets and approaches for clinical translation. This work offers essential insights for reprogramming platelets to shift their function from tumor promotion to tumor suppression, providing a foundation for the development of novel immunotherapeutic strategies and related research.

## Introduction

Platelets, the anucleate fragments derived from megakaryocytes, are classically recognized for their role in hemostasis and thrombosis [1]. However, accumulating evidence indicates they also critically contribute to cancer progression. Cancer patients often present with thrombocytosis and a hypercoagulable state, both of which correlate with poorer clinical outcomes. Indeed, individuals with elevated platelet counts face an increased risk of developing malignancies [2]. Through diverse mechanisms, platelets interact directly with tumor cells to drive epithelial-mesenchymal transition (EMT), foster resistance to therapy, and facilitate immune evasion [3, 4]. Moreover, by preserving the integrity of tumor vasculature, platelets prevent intratumoral hemorrhage and thereby support sustained tumor growth [5, 6].

Immunotherapy, designed to amplify the immune system’s capacity to recognize and eliminate malignant cells, has revolutionized cancer treatment, offering meaningful benefit to approximately 20-40% of patients. Nonetheless, its broader efficacy is curtailed by challenges such as off-target toxicities, suboptimal delivery of agents to the tumor bed, and complex manufacturing processes [7]. Recent studies have unveiled a dualistic role for platelets in cancer immunity: on one hand, they establish immunosuppressive niches that protect tumors; on the other, they directly modulate immune cell function [8]. Platelets can cloak tumor cells from immune recognition, inhibit effector lymphocyte activity, and shape a microenvironment that resists therapeutic intervention [9–12].

Against this backdrop, strategies aimed at modulating platelet function hold substantial promise for enhancing immunotherapeutic outcomes. Differing from existing reviews, this review aims to explore the multifaceted role of platelets in tumor-immune crosstalk, underscore their potential as targets in cancer immunotherapy, and explore emerging platelet-based therapeutic approaches with an eye toward clinical translation. We do hope to provide a roadmap for future research on reprogramming platelets from promoters of tumor into facilitators of anti-tumor immunity.

## Platelets in tumor initiation and progression

### Platelet-mediated promotion of tumor cell proliferation

Tumor cells release soluble activators like adenosine triphosphate that recruits and activates platelets within the tumor stroma. Once activated, platelets adhere to cancer cells via surface molecules including integrin-αIIbβ3 [13], P-selectin [13], and C-type lectin-like receptor 2 [14]. This direct physical interaction has been shown to accelerate the growth of hemangioendothelioma. Notably, knockdown of platelet integrin β3, which binds to galectin-3 on tumor cell, significantly abolishes platelet-induced tumor cell proliferation [4]. Beyond these contacts, platelets discharge a suite of growth-promoting cytokines and chemokines, such as tumor growth factor-β (TGF-β) [15, 16], vascular endothelial growth factor (VEGF) [17], platelet-derived growth factor (PDGF) [18], and platelet factor 4 (PF4) [19] that further fuel tumor expansion. Platelet-derived microparticles (PMVs) shed upon activation transfer oncogenic signals to lung cancer cells and upregulating cyclin D2 [20].

### Platelet-driven angiogenesis

In addition to directly stimulating tumor cell division, platelets are central to neovascular formation. They sequester and transport large quantities of VEGF, positioning them as the predominant source of this key angiogenic mediator in the circulation [21, 22]. Clinically, raised VEGF levels in cancer patients correlate closely with platelet counts [23]. Tumor-activated platelets also release PMVs that induce transcription of angiogenic factors in endothelial cells [20], while their α-granule contents rich in pro-angiogenic proteins amplify new vessel growth in cancers such as breast carcinoma via VEGF-dependent pathways [17]. Notably, anti-estrogen therapies like tamoxifen and its active metabolite 4-hydroxytamoxifen can impede this process by altering platelet reactivity and reducing their support of angiogenesis [24]. Moreover, platelets play a protective role in maintaining tumor vascular integrity, thereby preventing hemorrhage and sustaining perfusion within the growing mass [22]. Collectively, these findings highlight platelets as promising targets for anti-angiogenic strategies aimed at stalling tumor progression.

### Platelets promote cancer metastasis

Another well-known function of platelets in cancer progression is their ability to promote metastasis. At the primary tumor site, platelets actively induce EMT, endowing carcinoma cells with enhanced motility and invasiveness through interacting directly with tumor cells and releasing metastasis-promoting factors [6, 25–27]. In co-culture experiments, breast and colon cancer cells exposed to platelets acquire a spindle-shaped morphology, upregulate matrix metalloproteinase-9 (MMP-9) and downregulate epithelial proteins like E-cadherin. Mechanistically, platelet-derived TGF-β and NF-κB activators drive the transcription of EMT master regulators like *Snail1*, thereby fostering a more aggressive phenotype [6]. In vivo, pretreatment of tumor cells with platelets increases pulmonary colonization, confirming their role in seeding metastatic niches.

Once cancer cells intravasate, they face detachment-induced apoptosis (anoikis). Platelets counter this by stimulating YAP1 signaling pathways that promote survival [28]. Immediately upon entering the bloodstream, circulating tumor cells (CTCs) become coated with platelets, which buffer them against hemodynamic shear forces [29]. Disruption of key platelet receptors, such as integrin αIIbβ3 or P-selectin, impairs this cloaking effect and sensitizes CTCs to mechanical stress [13, 30]. Beyond mechanical protection, platelet cloaks also confer immune evasion by impairing natural killer (NK) cell recognition, thus promoting CTC metastasis both in vitro and in vivo [9, 31, 32]. Furthermore, platelets transfer adhesion molecules (e.g., CD61, CD42) onto the surface of CTCs, bolstering their ability to adhere to endothelium and arrest at distant sites [33, 34]. Platelet-released ATP engage endothelial P2Y_2_ receptors, transiently loosening intercellular junctions and facilitating transendothelial migration [35]. Finally, platelets also contribute to the establishment of metastatic niches. In the pre-metastatic niche, tumor-activated platelets secrete chemokines such as CXCL5 and CXCL7, which recruit granulocytes and remodel the local microenvironment to support incoming tumor cells [36]. Depletion of platelets markedly reduces lung colonization by tumor cells, underscoring their essential role in niche formation. Genetic or pharmacological inhibition of platelet receptors like glycoprotein VI (GP VI) [37], effectively disrupts the formation of metastatic microenvironments.

To sum up, platelets orchestrate multiple steps of the metastatic cascade, from EMT induction to CTC protection to metastatic niche formation. Future studies are needed to elucidate the molecular mechanisms by which platelets promote metastasis, particularly their interactions with CTCs and regulation of EMT-related pathways.

## Dual roles of platelets in cancer immunity

Emerging evidence shows that platelets can both undermine and bolster anti-tumor immunity, with their net effect hinging on the stage of disease, and receptor-ligand interactions. For instance, in early lesions, platelet-derived TGF-β may enforce cell-cycle arrest and induce tumor cell dormancy [37, 38], yet in advanced tumors it shields cancer cells from immune attack, and fosters an immunosuppressive tumor microenvironment (TME) [39]. This duality of platelets underscores their complexity in tumor immunity. To unpack these seemingly opposing effects, we organize our discussion into two sections: platelet-mediated immunosuppression and platelet-driven immune activation.

### Platelets in tumor-promoting immunity

#### Platelets protect CTCs from NK cell elimination

**Fig. 1:**
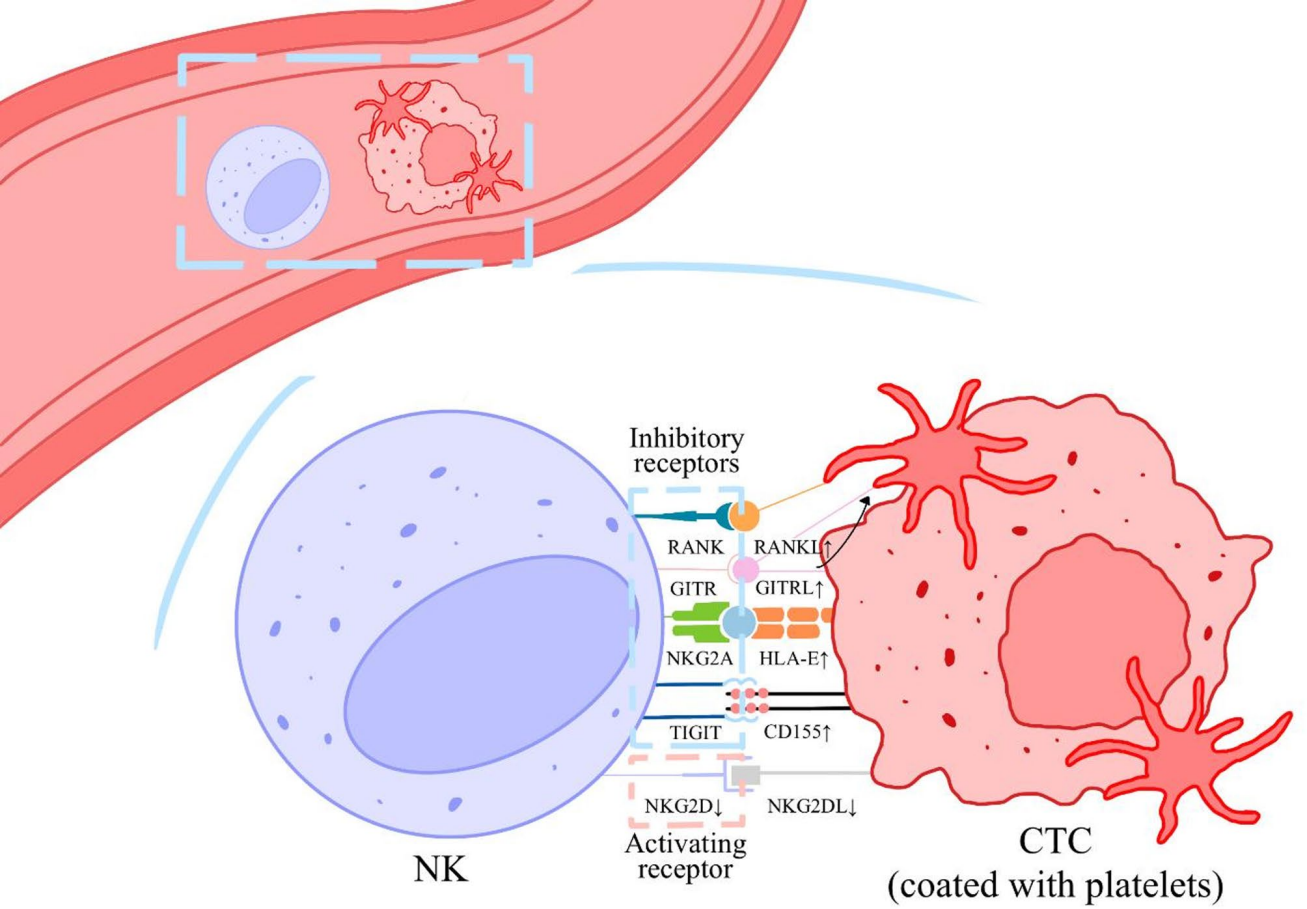
Platelets protect CTCs from NK cell elimination. Once tumor cells enter the bloodstream, platelets rapidly aggregate around them, forming a protective barrier that shields CTCs from NK recognition. This platelet encapsulation leads to upregulation of inhibitory receptor ligands, such as HLA-E on CTC, while simultaneously downregulating ligands for activating receptor like NKG2D. This dual modulation effectively inhibits NK cell-mediated tumor cells killing. Moreover, platelets exhibit increased expression of RANKL, which binds to the inhibitory RANK on NK cells, further suppressing their cytotoxic activity. Additionally, GITRL has been observed to transfer from tumor cells to the surface of platelets, further contributing to the immune evasion. CD, cluster of differentiation; CTC, circulating tumor cell; HLA, human leukocyte antigen; GITRL. glucocorticoid-induced TNFR-related protein ligand; NK, natural killer; NKG2, natural killer group 2; RANK, receptor activator of nuclear factor-kappa B; TIGIT, T cell immunoreceptor with Ig and ITIM domain

NK cells serve as critical guardians against hematogenous metastasis, eliminating CTCs through contact-dependent cytotoxicity regulated by a balance of activating and inhibitory receptors [40]. Platelet-mediated suppression of NK cell function primarily occurs through three interconnected mechanisms (Fig. 1). (1) Forming peri-tumoral aggregates that physically shield CTCs from NK cell-mediated cytolysis [9]. (2) Secreting immunosuppressive cytokines such as interleukin-10 (IL-10) and TGF-β to downregulate NK-activating receptors while upregulating inhibitory receptors [41, 42].

Following intravasation into circulation, CTCs immediately recruit platelets establish protective microaggregates that dampens NK cell functions [9]. It revealed that platelet-derived MHC class I molecules confer pseudo-self-characteristics to CTCs, effectively bypassing NK cell missing-self recognition mechanisms. This molecular camouflage results in marked reductions in both NK cell cytotoxicity and interferon-γ (IFN-γ) production, establishing a permissive environment for metastatic progression. Emerging evidence indicates that RGS18, a platelet-enriched regulator of G-protein signaling, promotes CTC immune evasion by activating the HLA-E: CD94-NKG2A immune checkpoint axis [41, 43]. Blocking NKG2A can improve NK cell activity in vitro and hinder CTC dissemination in vivo [41]. The TGF-β/NKG2D axis represents another key regulatory pathway, where platelet-derived TGF-β downregulates NKG2D expression on NK cells, thereby impairing cytotoxic granule exocytosis, target cell lysis, and IFN-γ production [42, 44]. This immunosuppressive effect can be abrogated through TGF-β neutralization. Furthermore, platelet-coating of CTCs induces proteolytic shedding of NKG2D ligands (NKG2DLs) from tumor cell surfaces, effectively reducing NK cell recognition efficiency [45].

TIGIT/CD155 immune checkpoint axis contributes significantly to platelet-mediated immune suppression. Tumor microenvironmental upregulation of CD155, the canonical TIGIT ligand, correlates with poor clinical outcomes and therapeutic resistance across multiple malignancies [46–48]. Mechanistically, platelet-tumor cell adhesion induces transcriptional activation of CD155 in CTCs, facilitating TIGIT-mediated NK cell exhaustion through sustained receptor-ligand engagement [49]. Disruption of TIGIT/CD155 interactions using neutralizing antibodies restores NK cell cytotoxic capacity and reduces metastatic dissemination in preclinical models.

Emerging evidence highlights the role of glucocorticoid-induced TNFR-related protein ligand (GITRL) in platelet-dependent immune evasion. Tumor cells exploit platelet-mediated GITR activation through two complementary mechanisms: direct translocation of platelet-derived GITRL to tumor membranes, and upregulation of platelet-constitutive GITRL expression during tumor-induced activation [50, 51]. This dual-source GITRL pool synergistically suppresses NK cell function through GITR co-stimulation [52]. Additionally, tumor-activated platelets upregulate membrane-bound receptor activator of nuclear factor-κ B ligand (RANKL) isoforms that directly impair NK cell effector functions, an immunosuppressive phenotype partially reversible through RANKL inhibition [53].

#### Platelets contribute to immunosuppressive TME

**Fig. 2:**
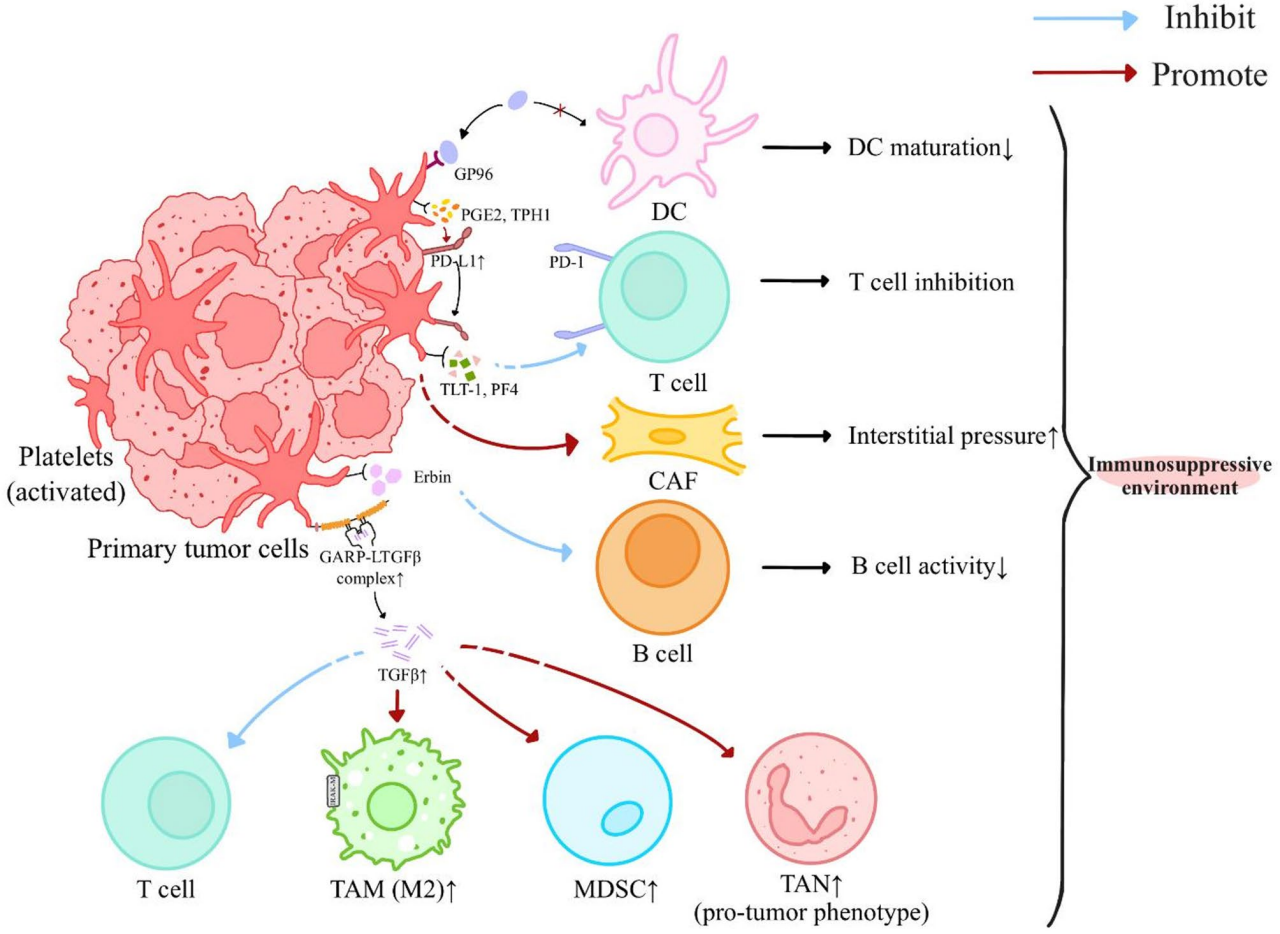
Platelets and TGF-β in shaping immunosuppressive TME. Tumor cells are found to transfer PD-L1 to the surface of platelets, thereby inhibiting T cell-mediated immune response. The level of PD-L1 is elevated by platelet releases, including PGE2 and TPH1. Additionally, TLT1, PF4, and Erbin have been demonstrated to impair the functions of T cells and B cells, respectively. Platelets also positively influence CAFs, causing increased interstitial pressure in tumor stroma. In addition, the release of active TGF-β from the GARP-LTGF-β complex on platelets is elevated, promoting pro-tumor cells such as M2 macrophages, MDSCs and TANs, while simultaneously inhibiting cytotoxic T cell, thus enabling immune exclusion. All these effects collectively contribute to an immunosuppressive TME. CAF, cancer associated fibroblasts; DC, dendritic cell; GARP, glycoprotein-A repetitions predominant; LTGF-β, latent transforming growth factor beta; GP, glycoprotein; MDSC, myeloid-derived suppressor cell; PD-L1, programmed cell death ligand 1; PF4, platelet factor 4; PGE2, prostaglandin E2; TAM, tumor-associated macrophage; TAN, tumor-associated neutrophil; TLT-1, TREM-like transcript 1; TME, tumor microenvironment; TPH1, tryptophan hydroxylase 1

The immunosuppressive milieu represents a major therapeutic barrier in cancer immunotherapy, with platelets serving as key mediators of this immunosuppressive niche through multiple synergistic mechanisms (Fig. 2).

Platelets exert multifaceted control over the programmed cell death ligand 1 (PD-L1) immune checkpoint system [54]. Tumor-platelet interactions facilitate bidirectional PD-L1 transfer through direct membrane protein exchange and platelet-mediated transcriptional upregulation of PD-L1 in tumor cells [55–57]. In non-small cell lung cancer (NSCLC), platelet-derived PD-L1 directly suppresses CD4+/CD8 + T cell activity [55]. Mechanistically, platelets promote PD-L1 by both contact-dependent and non-contact-dependent manners. platelet-secreted metabolites including tryptophan hydroxylase 1 (TPH1) and prostaglandin E2 (PGE2) synergistically enhance tumor PD-L1 expression through STAT3/NF-κB signaling activation, while pharmacological inhibition of these pathways enhances immune cell infiltration [58, 59]. In addition to promoting PD-L1 expression, platelet-derived PGE2 contributes to immunotherapy resistance through multiple mechanisms. For instance, PGE2 binds to EP2/EP4 receptors on T cells to impair mitochondrial oxidative phosphorylation and induce metabolic exhaustion. It also polarized tumor-associated macrophages (TAMs) toward an immunosuppressive M2 phenotype through COX-2/PGE2/PPARγ signaling. This cascade ultimately leads to T cell exhaustion and compromised anti-tumor immunity [60, 61]. Clinically, elevated PGE2 levels in platelets have been linked to poor immunotherapy outcomes in EBV-negative gastric cancer, whereas Epstein-Barr virus (EBV)-positive tumors with lower PGE2 exhibit better responses, highlighting the therapeutic potential of targeting platelet-PGE2 signaling [62].

Platelets also compromise dendritic cell (DC) functionality. DC activation induced by glycoprotein 96 (GP96) is restrained due to its interaction with GP96 receptors on platelets [63]. Platelets markedly inhibit pro-inflammatory cytokines production from DCs, resulting in anti-inflammatory phenotype of DCs and subsequently hinder T cell-mediated adaptive immune response [64]. Clinically, DCs derived from platelet-monocyte aggregates showed downregulated activation markers such as CD206 and CD80, and impaired antigen-presenting capacity in Human Immunodeficiency virus (HIV) patients [65]. Given the shared immunosuppressive features between HIV pathogenesis and cancer progression, platelets may exert comparable immunomodulatory effects on DC maturation in malignancy settings. Notably, the observed DC dysfunction in HIV provides mechanistic insights for understanding cancer-related immunosuppression, particularly in HIV-associated malignancies where platelet activation is frequently elevated. This cross-disease conservation highlights the potential of targeting platelet-DC interactions to restore anti-tumor immunity in immunocompromised populations.

Emerging evidence reveals platelet-derived substances modulate TME. TREM-like transcript 1 (TLT-1), a platelet protein, elevated in NSCLC platelets, induces CD8 + T cell dysfunction via CD3ε interaction [66]. Soluble TLT-1derived from platelets interacts with monocytes, initially activating them. However, this interaction subsequently triggers an immunosuppressive phenotype in monocytes, ultimately compromising the immune system [67]. Through PDGF-B signaling, platelets activate cancer-associated fibroblasts (CAFs) to drive extracellular matrix deposition and increase interstitial pressure [68–70]. Pharmacological PDGF-B inhibition not only downregulates CAF activation but also normalizes TME mechanical properties, suggesting combinatory potential with immunotherapies [68, 70].

#### Platelet-derived TGF-β negatively affects immune system

Platelet-secreted TGF-β is a pivotal cytokine that dampens anti-tumor immunity within the tumor microenvironment (Fig. 2). It directly inhibits CD8⁺ T-cell proliferation and effector functions [39], contributing to resistance against immunotherapies and correlating with poor clinical outcomes [71]. Moreover, TGF-β impairs the activity of T cell-recruiting bispecific antibodies, including the approved CD19×CD3 agent blinatumomab [72]. In thrombopoietin receptor agonist models, elevated platelet counts coincide with expansion of myeloid-derived suppressor cells (MDSCs), an axis abrogated by Smad2/3 knockdown and implicating platelets in MDSC activation via TGF-β [73].

TGF-β is synthesized as a latent complex requiring activation at the cell surface [74]. Glycoprotein-A repetitions predominant (GARP), encoded by *lrrc32*, anchors latent TGF-β on platelets, localizing its activation within tumors [75]. Although physiologic GARP-TGF-β signaling maintains peripheral tolerance, *lrrc32* amplification and overactive GARP-TGF-β signaling have been observed across multiple cancers, correlating with an immune-tolerant microenvironment and poor responses to immune checkpoint inhibitors (ICIs) [12, 75, 76]. Monoclonal antibodies targeting the GARP-TGF-β axis restore T-cell infiltration without broadly impairing host defenses, including IgA-mediated mucosal immunity [76–78]. Notably, platelet-specific deletion of *lrrc32* interferes intratumoral TGF-β activity and enhances anti-tumor immunity in melanoma and colon cancer models [79], and pharmacologic inhibition of platelet activation or TGF-β maturation further boosts chimeric antigen receptor T-cell (CAR-T) therapy and ICIs in preclinical studies [12, 79].

As stromal cell-derived TGF-β has been extensively characterized, future work should clarify the specific contributions of platelet-derived versus other cellular sources of TGF-β to these anti-tumor effects.

### Platelets in anti-tumor immunity

**Fig. 3:**
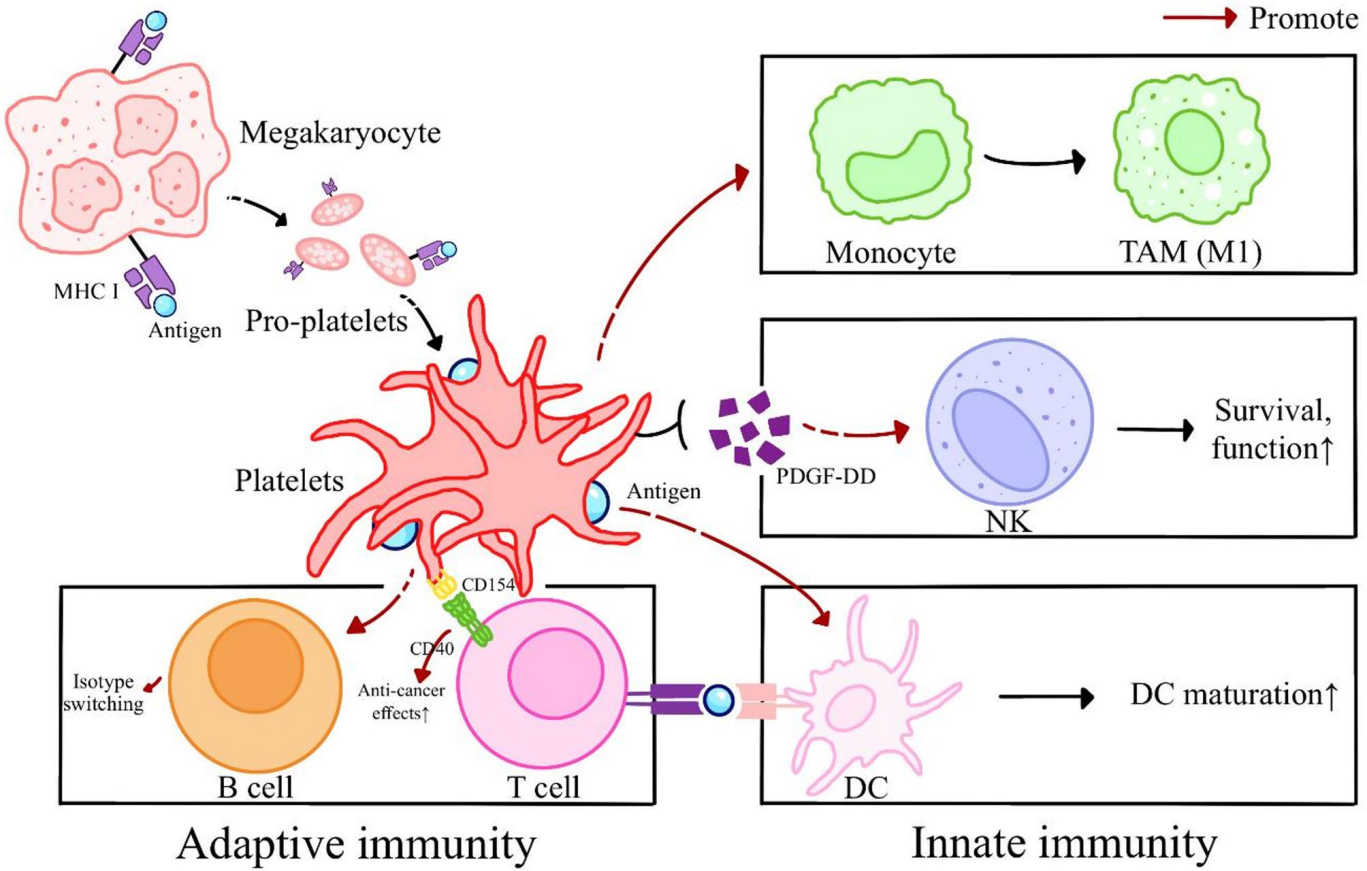
The role of platelets in anti-cancer immunity. Platelets act as a be a bridge between innate and adaptive immunity. They promote the differentiation of monocytes into anti-cancer M1 macrophages. Platelet-derived PDGF-DD supports the survival and function of NK cells. Interestingly, megakaryocytes and pro-platelets, the precursors of platelets, express MHC hI molecules, allowing them to recognize both exogenous and endogenous antigens. Platelets can shuttle antigens within the circulation and deliver them to DCs, facilitating DC maturation and subsequent cell activation, thereby eliciting an adaptive immune response. The expression of CD154 on platelets interacts with CD 40 on multiple immune cells, such as T cells, to enhance their anti-cancer effects. Additionally, platelets have been shown to induce the isotype switching of B cells, bolstering their ability to combat infections and malignancies. CD, cluster of differentiation; DC, dendritic cell; MHC, major histocompatibility complex; NK, natural killer; PDGF, platelet-derived growth

Despite their well-documented immunosuppressive functions, platelets can also bolster anti-tumor immunity through several complementary mechanisms. By releasing chemokines, cytokines, and surface ligands, they promote M1 polarization of macrophages, drive DC maturation, facilitate B-cell isotype switching, and enhance T-cell responses both in vitro and in vivo (Fig. 3) [80, 81]. For example, PDGF-DD supports NK cell survival and cytotoxicity [82, 83], with elevated PDGF-DD-activated NK cells correlating with improved prognosis in low-grade glioma patients [84]. Platelets likewise induce a proinflammatory phenotype in monocytes [85], while co-culture with DCs triggers their differentiation into mature, antigen-presenting cells that more efficiently stimulate lymphocyte proliferation [86, 87]. In vivo, platelets shuttle tumor antigens to splenic DCs, precipitating robust CD8⁺ T-cell responses [88].

Megakaryocytes, the progenitors of platelets, have been shown to process and present endogenous and exogenous antigens on MHC-I, transferring antigen-loaded MHC-I complexes to nascent platelets and suggesting a direct antigen-presentation role for platelets themselves [89]. Consistent with this, platelet depletion diminishes cytotoxic T-lymphocyte infiltration in tumors, an effect reversible by platelet transfusion [90]. Furthermore, platelet-derived microparticles transfer integrin β₃ to T cells, enhancing their activation and offering a potential explanation for the clinical failure of the integrin inhibitor cilengitide [91]. Supplementing T-cell cultures with human platelet lysate fosters sustained proliferation and durable anti-tumor activity compared with standard sera [92].

Platelet surface CD154 provides a potent co-stimulatory signal: it engages CD40 on T cells, B cells, DCs, and macrophages to amplify proinflammatory cytokine production and augment anti-tumor responses [93, 94]. In murine models of hepatocellular carcinoma arising in nonalcoholic fatty liver disease, CD154-positive platelets are essential for limiting tumor growth and genetic or pharmacologic loss of CD154 abolishes this effect, whereas transfusion of CD154-expressing platelets restores it [95]. Platelet-rich fibrin (PRF) implants, rich in immunomodulatory factors including CD154, have shown promise as adjuvants: PRF patches inhibit tumor cells proliferation in vitro and prolong survival in glioma-bearing mice [96]. Analysis revealed that attenuated Tregs infiltration and migration. Mechanistically, CD154 plays a key role in PRF-mediated Tregs inhibition, and blocking CD154 allows Tregs to regain their migration ability [97].

Finally, although TGF-β is predominantly immunosuppressive in established tumors, it can exert tumor-suppressive effects in early carcinogenesis by enforcing cell-cycle arrest and dormancy [37, 38, 98]. Thus, platelets’ capacity to release TGF-β, alongside their antigen-presenting and co-stimulatory functions, underscores their dualistic role as both inhibitors and enhancers of anti-tumor immunity. Further research must delineate the contextual cues that tip this balance, with the goal of harnessing platelets’ immune-activating potential while mitigating their immunosuppressive activities.

## Reprogramming platelets in immunotherapy

Given their multifaceted contributions to tumor growth, angiogenesis, metastatic dissemination, and the establishment of an immunosuppressive microenvironment, platelets represent an attractive target for enhancing cancer immunotherapy [3, 8]. Beyond strategies aimed at blocking platelet-tumor interactions, recent efforts have focused on harnessing platelets as bio-inspired drug carriers. Their innate tropism for tumor cells, long circulation half-life, and excellent biocompatibility make platelets ideal vectors for delivering therapeutics directly to the tumor bed. Approaches under investigation include loading cytotoxic or immunomodulatory agents into autologous platelets, as well as cloaking nanoparticles with platelet membranes to exploit platelet surface receptors for tumor targeting [99, 100]. Several of these platelet-based delivery systems have advanced to early-phase clinical evaluation (Table 1).

### Anti-platelet triggers immunotherapy improvement

A 20-year study reported that long-term, low-dose aspirin reduces both the incidence and the mortality of colon cancer [101], spurring interest in antiplatelet agents as adjuncts to cancer therapy [102]. In preclinical models, drugs that inhibit platelet function, such as aspirin, clopidogrel, and low-molecular-weight heparin (LMWH), not only limit metastatic spread, but also remodel the TME to favor immune attack [39, 103]. By disrupting platelet support for tumor vasculature, these agents increase vessel permeability, improving intratumoral delivery of therapeutics while permitting dose reductions and minimizing systemic toxicity [5, 104, 105]. While accumulating evidence supports the synergistic effect of aspirin with immunotherapy, the underlying mechanisms remain debated. On one hand, aspirin may act through platelet-dependent pathways: by inhibiting platelet activation via COX-1 blockade, it reduces the release of immunosuppressive factors that otherwise dampen T cell cytotoxicity [106]. On the other hand, aspirin’s direct inhibition of tumor-intrinsic COX-associating signaling could reprogram the immunosuppressive TME, promoting dendritic cell maturation and CD8 + T cell infiltration [107]. Resolving this dichotomy requires innovative experimental designs. Future studies should employ platelet-specific knockout models to dissect platelet contributions. Ultimately, a dual-target strategy that simultaneously blocks platelet immunosuppression and tumor cell intrinsic pathways may maximize the benefits of aspirin in combination immunotherapy.

Combining antiplatelet therapy with ICIs or adoptive cell transfer therapy (ACT) yields synergistic benefits. For example, aspirin and clopidogrel treatment enhances the anti-tumor efficacy of anti-PD-1 antibodies [39] or CAR-T therapy [79]. Similarly, combining low molecular weight heparin (LMWH) with ACT significantly inhibited cancer progression and metastasis while enhancing the trafficking of intratumoral T cells and M1 macrophages and reducing the infiltration of M2 macrophages and Tregs, compared to using LMWH or ACT alone. A similar outcome was also observed when LMWH was combined with anti-PD-1 antibodies [103, 108]. Notably, as thrombocytosis is common in cancer patients and associated with poor survival, anti-platelet therapy has the potential to relieve thrombotic complications while maintaining its anti-cancer activity [109].

Beyond conventional drugs, platelet-targeted nanotherapeutics are emerging. A nanoparticle combining a platelet inhibitor, NO, with paclitaxel disrupts platelet-tumor interactions, enhances leukocyte infiltration, and reprograms the microenvironment toward an immune-supportive state [110]. A perfluorocarbon-based nanoparticle that selectively impairs platelet function in tumor vessels achieves 90% tumor growth inhibition when used with anti-PD-L1 antibodies. Notably, all the components used to construct it have been widely employed in clinical practice, making its application in the real world more feasible [111].

Targeting specific platelet-derived immunosuppressive mediators further potentiates immunotherapy. Inhibition of PDGFRα/β [10], VEGF [112], and TPH [59], enhances checkpoint blockade and restrains tumor progression. TGF-β has three isoforms: TGF-β1 functions mainly in malignancies while TGF-β2 and TGF-β3 are more involved in neovascular development. Selective inhibition of TGF-β1 combined with anti-PD-1 antibodies elicits robust anti-tumor responses and survival benefit in resistant murine tumor models without cardiotoxicity [113], and similar synergy is seen when pairing TGF-β1 blockade with cancer vaccines [11]. In a study involving Hodgkin lymphoma patients, through transfusion of TGF-β receptor-negative T cells, 4 out of 7 patients achieved complete remission, with two patients remaining in remission for more than four years [114]. A phase 1 trial using CAR-T cells engineered to silence TGF-β receptor for treating metastatic castration-resistant prostate cancer was completed, further verifying the feasibility of its application in clinical [115]. Several similar experiments are currently underway. Because platelets cloak circulating tumor cells and neutralize NK-cell activity via TGF-β, nanoparticle systems that targeting platelet-derived TGF-β have been developed. Albumin nanoparticles loaded with perfluorotributylamine selectively disrupt platelet function in tumor vessels, restoring NK-mediated clearance and reducing lung and liver metastases in multiple models [116]. It showed great potential in enhancing NK-mediated surveillance, leading to attenuated lung and liver dissemination in multiple metastasis models. Other TGF-β neutralizing paradigms similarly revive NK cytotoxicity and sensitize “cold” tumors to immunotherapy [117, 118].

Platelet inhibition has shown remarkable anti-cancer activity whether used alone or in combination with immunotherapy. Despite strong preclinical evidence that platelet inhibition can augment immunotherapy, the clinical translation of these strategies is impeded by a lack of tools to selectively target tumor-associated platelets. Lacking techniques that precisely target tumor-associated platelets impedes their application in clinical practice. Well-designed clinical trials are urgently needed to validate whether combining antiplatelet agents can potentiate immunotherapy in the real world.

### Leveraging platelets to enhance immunotherapy efficacy

#### Platelet-based nanomedicine

The propensity of platelets to aggregate within the tumor vasculature is increasingly exploited for therapeutic purposes. Mechanistically, platelet activation in tumors is driven by cancer cell-derived tissue factor, thrombin [119], and adenosine diphosphate [120], which engage PARs and P2Y12 receptors to trigger αIIbβ3 integrin activation and fibrinogen-mediated crosslinking. Activated platelets binds to tumor cells by integrin-αIIbβ3 [13], P-selectin [13], and C-type lectin-like receptor 2. Harnessing platelets’ innate tumor-homing properties and biocompatibility, researchers have begun cloaking nanoparticle cores with platelet membranes (PMs) to create biomimetic delivery vehicles that evade immune clearance and preferentially accumulate in the tumor microenvironment [121]. These PM-coated nanocarriers exploit P-selectin-mediated adhesion to neoplastic endothelium and even home to sites of bone metastasis, making them versatile platforms for a range of malignancies, such as multiple myeloma.

Utilizing PMs as long-range delivery system for biomimetic nanomedicines, has become a potential strategy for cancer treatment, enhancing uptake by tumor cells and attenuating toxicity in non-cancer sites [100]. PM-encapsulation of the toll-like receptor agonist R848 prolongs its retention within colorectal tumors, lowering the required dosage while potently activating antigen-presenting cells and driving CD8⁺ T-cell infiltration to induce tumor regression [122]. PM-coated anti-cancer drugs can also be used after tumor resection to prevent dissemination and recurrence by utilizing their property to aggregate at wound site. In the postoperative setting, hybrid membranes derived from platelets and neutrophils have been used to deliver R848 alongside CD47 blockade to surgical wound beds, skewing macrophages from an M2 to M1 phenotype, stimulating dendritic cell maturation, and preventing residual tumor recurrence [123].

Combining chemotherapy with immunotherapy often enhances anti-tumor efficacy but at the cost of systemic toxicity [124]. To address this, increasing the targeting of drugs is urgently needed to benefit more cancer patients. PM-coated nanoparticles carrying both sorafenib and anti-PD-1 antibodies achieve synchronized delivery to hepatocellular carcinoma cells, increasing drug uptake, minimizing off-target effects, and directly activating cytotoxic T cells for a superior therapeutic index [125]. Similarly, platelet-derived extracellular vesicles loaded with doxorubicin have been shown to capture circulating tumor cells, induce immunogenic cell death, and synergize with anti-PD-L1 therapy [126].

Emerging modalities such as photodynamic therapy (PDT) and ferroptosis induction can also benefit from PM coating. Although these therapies have gotten much attention by specifically killing tumor cells, their shortcomings like low response rates caused by low permeability and hypoxia in TME impede their application. To overcome these challenges, PMs have been designed as a delivery system to enhance drug concentration. Photosensitizer-loaded PM nanoparticles combined with anti-PD-1 antibodies enhance intratumoral immune infiltration and achieve greater tumor control than either modality alone, without detectable reproductive toxicity [127]. Hybrid platelet-photothermal nanodrugs target aberrant tumor vasculature; upon laser irradiation, they generate localized hyperthermia that both damages vessels and triggers PM vesicle release, deepening nanoparticle penetration, amplifying immunogenicity, and producing robust therapeutic effects across multiple mouse and xenograft models [128]. Prussian blue cores functionalized with PD-L1 and tumor-targeting aptamers and enveloped by PMs selectively accumulate in tumors and inflammatory sites, leading to potent photothermal ablation and in vivo activation of anti-tumor immunity [129]. PMs are also utilized to carry ferroptosis-inducing agents to evade immune clearance and concentrate at metastatic niches, reprogramming macrophages toward an M1 phenotype and driving iron-dependent tumor cell death [130].

Although PM-based nanomedicines hold promise for both early- and late-stage cancers, challenges remain in sourcing sufficient platelets and establishing standardized, scalable manufacturing processes. Continued innovation in platelet isolation, membrane purification, and nanoparticle assembly will be critical to translate these elegant biomimetic platforms into viable clinical therapies.

#### Engineered platelets

Engineered platelets exploit the innate tumor- and injury‐homing abilities of native thrombocytes by decorating them with immunotherapeutic agents, thereby concentrating treatment at disease sites while sparing healthy tissues [131]. For instance, platelets conjugated with anti-PD-1 antibodies traffic to bone marrow when administered intravenously; once in situ, they release ICIs, dramatically boosting T‐cell activation, cytokine production, and survival in a murine leukemia model [132]. Interestingly, the combination of IL-2 and anti-PD-1 antibodies has been demonstrated to influence the differentiation of T cell, leading to the generation of a distinct isotype of T cell, which resembles highly functional effector CD8^+^ T cells [133]. Building on this principle, a phase I study is underway combining platelet‐coupled IL-2 and anti-PD-1 therapy in patients with advanced solid tumors.

Following surgical resection, residual tumor cells often evade detection and seed metastases. Platelets loaded with anti-PD-1 or anti-PD-L1 antibodies home to wound sites and tumor vasculature, where they release PMVs bearing ICIs [134, 135]. In preclinical models, this strategy increased local drug concentration tenfold over free antibody infusion, reprogrammed the tumor microenvironment, and prevented both recurrence and the outgrowth of occult metastases [136]. Natural platelets modified with ICIs are also expanded to apply in other local treatments including thermal ablation, PDT, and high-density ultrasound ablation [137]. Platelets dual-labeled with iron oxide nanoparticles (for photothermal therapy) and anti-PD-L1 antibodies achieved synergistic tumor control and enhanced CD4⁺ and CD8⁺ T-cell infiltration following surgical removal of residual disease [138]. Combining engineered platelets with CAR-T cells also overcomes the immunosuppressive milieu that hampers adoptive cell therapies in solid tumors. Upon platelet activation, released PD-L1 PMVs remodel the tumor bed, facilitating CAR-T-cell infiltration and anti-tumor activity [139]. Additionally, platelets co-loaded with chemotherapeutics drugs [135], or immunostimulatory ligands [140] have demonstrated durable prevention of tumor relapse and improved survival by sustaining T-cell–mediated anti-tumor immunity.

Innovations to enhance specificity include fusing platelets with targeting proteins [141, 142]. platelets decorated with a fusion of truncated tissue factor and RGD peptide (tTF-RGD) selectively induce thrombosis in tumor vessels, recruiting more engineered platelets and amplifying local drug delivery without harming normal vasculature [142]. In another advance, asymmetrical urease functionalization endows platelets with self-propulsion in urea-rich environments, targeting tumors of the urinary tract [141]. Despite these promising results, standardized protocols for loading immune agents onto platelets remain limited, and challenges in large-scale production and quality control must be overcome before widespread clinical adoption. Nonetheless, the ability of engineered platelets to home to both primary and metastatic sites, deliver payloads with high precision, and synergize with existing immunotherapies offers a versatile platform for both early-stage adjuvant therapy and advanced cancer treatment.

In summary, traditional antiplatelet agents, PM-coated nanomedicines, and engineered platelets have each been shown to substantially enhance the efficacy of cancer immunotherapies, offering novel avenues to improve treatment outcomes and extend survival across disease stages. However, platelet-based interventions remain in their infancy. Critical challenges, including scalable platelet sourcing, standardized manufacturing, and precise targeting of tumor‐associated platelets, must be addressed, and mechanistically informed strategies developed, before these promising approaches can be translated into routine clinical practice.

#### The potential risks of platelet suppression

Although several platelet-targeting agents have shown excellent efficacy in non-oncologic diseases, their use in cancer treatment, especially in combination with immunotherapy, poses notable risks that must be carefully considered. Cancer patients, who are often elderly and have multiple comorbidities, represent a particularly vulnerable population in whom treatment regimens must be cautiously selected.

One major concern is the essential role of platelets in hemostasis and coagulation. Consequently, platelet inhibition significantly increases the risk of hemorrhagic events. For example, long-term aspirin use was associated with a 60% increase in gastrointestinal bleeding risk in a study with a mean follow-up of 4.7 years [143]. While thrombocytosis is commonly observed in cancer patients, others may present with thrombocytopenia or anemia that render platelet suppression particularly hazardous.

Beyond bleeding risks, the inhibition of platelets or platelet-derived factors can also provoke serious cardiovascular events. Celecoxib, for instance, has been implicated in promoting the formation of calcific nodules, thereby contributing to aortic valve calcification [144, 145]. Similarly, TGF-β, a multifunctional cytokine critical for homeostasis, has been associated with cardiovascular toxicity when broadly targeted. Pan-TGF-β antibodies induced cardiotoxicity in several preclinical models [146], and BMS-986,260, a small-molecule TGF-β receptor I inhibitor, was ultimately discontinued in clinical development due to cardiovascular safety concerns, despite its promising anti-tumor efficacy in combination with anti-PD-1 antibodies in murine models of colorectal cancer [147, 148].

Gastrointestinal toxicity is another frequent complication. In a randomized controlled trial involving 505 participants, nearly all patients receiving antiplatelet agents experienced varying degrees of digestive tract injury, with combination therapy causing more severe damage than monotherapy with aspirin or clopidogrel [149]. These concerns are particularly relevant for patients with gastrointestinal malignancies, who should undergo thorough evaluation before initiating antiplatelet-based treatments.

Finally, antiplatelet medications especially acetylsalicylates, may cause allergic reactions including skin rashes and bronchospasm, posing extra caution for patients prone to hypersensitivity when they are in combination with immunotherapy [150]. In light of these potential complications, it is crucial to consider strategies that enable selective targeting of tumor-associated platelets or the tumor microenvironment, rather than relying on systemic platelet inhibition. Such approaches may enhance therapeutic precision while minimizing off-target effects and treatment-related toxicity.

## Platelets in diagnosis and prediction of patients received immunotherapy

Because platelets avidly interact with tumor cells, they internalize tumor-derived proteins and RNAs, and their own transcriptomic and proteomic profiles shift in the presence of malignancy [151]. These platelets can therefore serve as a minimally invasive “liquid biopsy” to detect early or otherwise asymptomatic cancers and to monitor disease progression [152, 153].

In comparative analyses of pre-treatment blood samples from lung and pancreatic cancer patients versus healthy controls, platelets from cancer patients exhibited characteristic changes in volume, RNA content, and protein expression. A diagnostic model based on these platelet parameters distinguished lung cancer patients from healthy individuals with an area under the ROC curve (AUC) of 0.887, and achieved similar accuracy in pancreatic cancer (AUC = 0.827) [154]. As the central player in cancer progression, platelets offer a potential all-in-one platform. Best et al. reported that the platelet RNA profiles were altered in cancer patients. The diagnosis, localization, and companion diagnostics of cancer are achieved by sequencing platelets [155]. This group later designed an intelligence-enhanced algorithm based on altered platelet RNA, independently discriminating NSCLC patients from benign inflammatory conditions [156]. Similarly, across a cohort of 1,448 samples, platelet RNA profiling achieved high sensitivity and specificity for cancer screening and tumor localization [151]. Beyond nucleic acids, the level of platelet-derived releases such as GITRL [51], GARP [12], PF4 [157], and VEGF [21] alter parallel to cancer progression, offering additional monitoring tools.

Platelet biomarkers also hold promise for forecasting patient responses to ICIs. Platelet PD-L1 acquired from tumor cells correlates with suppression of CD4⁺ and CD8⁺ T-cell function, and a computational model measuring platelet PD-L1 load outperforms traditional histological PD-L1 scoring in predicting ICI responsiveness in NSCLC [55]. A predictive model including gene markers related to platelets and M2 macrophages is developed to stratify patients by likely benefit from immunotherapy [158].

Patients with increasing platelet count or venous thromboembolism (VTE) tend to have poor outcomes [15, 159]. Tumor-associated thrombotic event is the second cause of death in cancer patients [160, 161], further linking altered platelets to poor prognosis. However, little is known about TE in immunotherapy. TE is associated with a significant deterioration in survival in a retrospective cohort study with 228 melanoma patients receiving ICIs [162]. Other platelet-derived factors, such as TLT-1, have been linked to reduced overall survival in NSCLC patients [66]. Likewise, elevated PF4 levels in malignant pleural effusions signal diminished T-cell function and poor prognosis [157]. The platelet-to-lymphocyte ratio (PLR) is another readily available metric [163–165]. in metastatic NSCLC treated with nivolumab, a high PLR independently portends lower response rates and shorter overall survival [166]. Our previous research found that the PLR is associated with the efficacy of tumor immunotherapy, with higher ratios correlating with poorer outcomes [167]. Meta-analysis also revealed higher PLR is related to undesirable prognosis in NSCLC patients receiving ICIs [168, 169].

Platelet-based liquid biopsy is less invasive and more accessible to obtain compared to pathological biopsy. Hence, platelets are emerging as promising biomarkers for screening and monitoring, and for discriminating patients who are more likely to benefit from immunotherapy from cancer patients with limited efficacy. To transition these assays into clinical practice, large multicenter studies are needed to validate their sensitivity, specificity, and reproducibility across diverse tumor types and treatment regimens. Placing platelet diagnostics alongside existing biomarkers may enable truly personalized therapeutic strategies, ensuring that patients most likely to benefit from immunotherapy are accurately identified.

## Conclusions and future directions

Platelets are now recognized as central orchestrators of tumor progression, metastasis, angiogenesis, and immune regulation. Specifically, this review emphasizes their dualistic roles in modulating anti-tumor immunity and highlights multiple strategies for harnessing or inhibiting platelets to improve cancer immunotherapy [79, 170].

While platelets are essential defenders in innate immunity, within the TME they often promote immune evasion. They impair NK cell surveillance of CTCs, suppress T cell and dendritic cell function, and facilitate immunosuppressive myeloid infiltration and macrophage polarization [12, 41, 79]. Although the majority of evidence suggests platelets support tumor growth, some studies report contradictory findings [86, 90, 95], reflecting their context-dependent roles within the TME. The interplay between platelets and tumor cells also modulates the level of ICs, which further results in immune escape [56, 57]. In contrast, platelets and their secretions, such as PDGF-DD [82], enhance the anti-cancer ability of the immune system, suggesting their possibility of inducing immune cell pro-inflammatory phenotypes. Additionally, our ongoing work indicates that platelets may interact with tumor cells through the HMGB1/LRRC32 signaling axis, thereby influencing the tumor immune microenvironment. However, the specific mechanisms require further investigation.

Current therapeutic strategies include traditional antiplatelet agents and platelet-targeted drug delivery systems. The major limitation of conventional antiplatelet therapy is increased bleeding risk due to non-selective inhibition. Targeted approaches, particularly those blocking TGF-β, have shown promising synergy with immunotherapies in both preclinical and clinical studies [171, 172].

Novel platelet-based therapy, including PM-conjugated nanodrugs and engineered platelets that utilize the unique features of platelets have been developed. Since platelets are recognized as ‘self’ cells, they are able to escape the immune elimination and therefore prolong the retention in blood. Recently, tumor cells have been observed to recruit and activate platelets. By taking advantage of this feature, drug delivery systems based on platelets can be optimized to increase drug accumulation in tumors while reducing toxicity at nontumor sites compared to inorganic materials. Notably, the residual microtumor lesion and undetectable CTCs are the main reasons for dissemination and metastasis after tumor resection. Platelets can aggregate to in situ tumor cells and CTCs, showing promising efficacy in neoadjuvant therapy [135, 139]. In addition, T cells for CAR-T therapy cultured in human platelet lysate exhibited durable higher proliferation and tumor-killing ability [92]. However, these approaches remain largely preclinical, with no current human trials validating efficacy.

An additional challenge lies in platelet stability and storage. Currently, plateletbased drug formulations are typically freshly prepared; however, in clinical practice, stored platelets are more commonly used. It is crucial to determine whether stored platelets can maintain their activity and biocompatibility. Indeed, stored thrombocytes showed interfered pharmacokinetics, which may also affect the efficacy of engineered platelets and platelet-coated nanomedicines [173, 174]. Thierry Burnouf et al. demonstrated the characterization and bioactivity of cooling doxorubicin-loaded platelets remaining after preserving at -80℃ [175], which may provide a direction for translational feasibility of platelet-based medicine. The industrialization of platelet products is also one of the problems to be solved. Clinical application requires large quantities of platelets; therefore, attentions must be paid to the source of platelets and batch consistency Furthermore, transfusion of allogeneic platelets may cause immunological rejection. Therefore, there is a need to develop standardized methods for plateletbased drug preparation.

Platelets are also emerging as valuable tools for diagnosing and predicting outcomes in cancer patients receiving immunotherapy. Due to active crosstalk with tumor cells, platelets can acquire PD-L1, potentially reflecting intratumoral expression levels [55]. Liquid biopsies based on platelet biomarkers are less invasive and easier to perform than tissue biopsies, with growing evidence suggesting high predictive value for immunotherapy response. Nonetheless, large, multicenter validation studies are necessary to confirm their clinical utility.

Altogether, platelets contribute to tumor growth, metastasis, and the formation of immunosuppressive milieu. Combining platelet inhibition with immunotherapy could increase the immune cell infiltration and reverse the immunosuppressive TME. The strong targeting of platelet-based nanomedicine and engineered platelets is also proposed. Future research should focus on clarifying the molecular mechanisms underlying platelet-tumor interactions and conducting robust clinical trials to confirm the safety, efficacy, and translational potential of platelet-based strategies.

**Table 1:** Representative clinical trials of platelet-based intervention to improve immunotherapy

Therapy modality	Agents	Modes of intervention	Cancer types	Study phase	Status	ClinicalTrials. gov reference
Anti-platelet	ASA	In combination with Pembrolizumab (anti-PD-1 antibody) and Ipilimumab (anti-CTLA-4 antibody)	Metastatic or unresectable melanoma	II	Completed	NCT03396952 [176]
		In combination with Avelumab (anti-PD-L1 antibody) and Lansoprazole	TNBC	II	Withdrawn	NCT03794596
		In combination with Avelumab (anti-PD-L1 antibody) and Lansoprazole	TNBC	II	Not yet recruiting	NCT04188119
		In combination with BAT1306 (anti-PD-1 antibody)	microsatellite instability-high/deficiency mismatch repair) colorectal cancer	II	Recruiting	NCT03638297 [177]
		In combination with Rintatolimod (TLR3 agonist) with or without IFN-α2b	Prostate cancer^a^	II	Recruiting	NCT03899987
		In combination with P1101 (ropeginterferon α-2b)	Polycythemia vera	III	Recruiting	NCT06002490
		In combination with Serplulimab (anti-PD-1 antibody) and Chemotherapy	Untreated extensive-stage SCLC	II	Not yet recruiting	NCT06554535
		In combination with immunomodulatory cocktail (Vitamin D, Cyclophosphamide and Lansoprazole), Pembrolizumab and Radiation.	Advanced and refractory cervical cancer, endometrial carcinoma or uterine sarcoma	II	Completed	NCT03192059 [178]
	ASA or celecoxib	In combination with Nivolumab (anti-PD-1 antibody), Radiation and Cyclophosphamide or Ipilimumab	Advanced or metastatic incurable solid tumors	I	Completed	NCT03728179 [179]
	ASA and clopidogrel	In combination with Pembrolizumab	Recurrent or metastatic HNSCC	I	Recruiting	NCT03245489
	ASA	In combination with Atezolizumab (anti-PD-L1 antibody) and Bevacizumab	Recurrent ovarian cancer	II	Completed	NCT02659384
Engineered platelet	Platelet-coupled IL-2	In combination with PD-1 inhibitors	Advanced malignant solid tumors	I	Recruiting	NCT05829057

a: in the neoadjuvant condition

Abbreviations: ASA, aspirin; CTLA-4, cytotoxic T lymphocyte antigen 4; HNSCC, squamous cell carcinoma of head and neck; IL, interleukin; PD-1, programmed cell death 1; PD-L1, programmed cell death 1 ligand 1; SCLC, small cell lung cancer; TLR, toll-like receptor; TNBC, triple-negative breast cancer

## Data Availability

No datasets were generated or analysed during the current study.

## Abbreviations

ACT: Adoptive cell therapy
AUC: Area under the ROC curve
CAF: Cancer-associated fibroblast
CAR-T: Chimeric antigen receptor T-cell immunotherapy
CTC: Circulating tumor cell
DC: Dendritic cell
EBV: Epstein-Barr virus
EMT: Epithelial-mesenchymal transition
GARP: Glycoprotein-a repetitions predominant
GITRL: Glucocorticoid-induced TNFR-related protein ligand
GP VI: Glycoprotein VI
GP96: Glycoprotein 96
HIV: Human Immunodeficiency virus
HLA-E: Human leukocyte antigen E
ICI: Immune checkpoint inhibitor
IFN-γ: Interferon-γ
LMWH: Low molecular weight heparin
MDSC: Myeloid-derived suppressor cell
MHC I: Major histocompatibility complex I
NK: Natural killer
NSCLC: Non-small cell lung cancer
PD-1: Programmed cell death 1
PDGF: Platelet-derived growth factor
PD-L1: Programmed cell death ligand 1
PDT: Photodynamic therapy
PF4: Platelet factor 4
PGE2: Prostaglandin E2
PLR: Platelet to lymphocyte ratio
PM: Platelet membrane
PMV: Platelet-derived microparticle
PRF: Platelet-rich fibrin
RANKL: Receptor activator of nuclear factor-kappa B ligand
TAM: Tumor-associated macrophage
TGF β: Transforming growth factor β
TIGIT: T cell immunoreceptor with Ig and ITIM domain
TLT-1: TREM-like transcript 1
TME: Tumor environment
TPH1: Tryptophan hydroxylase 1
Treg: Regulatory T cell
VEGF: Vascular endothelial growth factor
VTE: Venous thromboembolism
